# A Three–MicroRNA Signature as a Potential Biomarker for the Early Detection of Oral Cancer

**DOI:** 10.3390/ijms19030758

**Published:** 2018-03-05

**Authors:** Yi-An Chang, Shun-Long Weng, Shun-Fa Yang, Chih-Hung Chou, Wei-Chih Huang, Siang-Jyun Tu, Tzu-Hao Chang, Chien-Ning Huang, Yuh-Jyh Jong, Hsien-Da Huang

**Affiliations:** 1 Department of Biological Science and Technology, National Chiao Tung University, Hsinchu City 300, Taiwan; b8703126@gmail.com (Y.-A.C.); chchou23@gmail.com (C.-H.C.); loveariddle.bi96g@g2.nctu.edu.tw (W.-C.H.); yjjongnctu@gmail.com (Y.-J.J.); 2 Department of Medical Research, Hsinchu Mackay Memorial Hospital, Hsinchu City 300, Taiwan; 3 Department of Medicine, Mackay Medical College, New Taipei City 252, Taiwan; 4467@mmh.org.tw; 4 Department of Obstetrics and Gynaecology, Hsinchu Mackay Memorial Hospital, Hsinchu City 300, Taiwan; 5 Mackay Junior College of Medicine, Nursing and Management College, Taipei 112, Taiwan; 6 Department of Medical Research, Chung Shan Medical University Hospital, Taichung 402, Taiwan; ysf@csmu.edu.tw; 7 Institute of Medicine, Chung Shan Medical University, Taichung 402, Taiwan; cshy049@csh.org.tw; 8 Institute of Bioinformatics and Systems Biology, National Chiao Tung University, Hsinchu City 300, Taiwan; mist0205@gmail.com; 9 Graduate Institute of Biomedical Informatics, Taipei Medical University, Taipei 110, Taiwan; kevinchang@tmu.edu.tw; 10 Department of Internal Medicine, Division of Endocrinology and Metabolism, Chung Shan Medical University Hospital, Taichung 402, Taiwan; 11 Graduate Institute of Clinical Medicine, College of Medicine, Kaohsiung Medical University, Kaohsiung 807, Taiwan; 12 Departments of Pediatrics and Laboratory Medicine, Kaohsiung Medical University Chung-Ho Memorial Hospital, Kaohsiung 807, Taiwan

**Keywords:** miRNA, biomarker, oral cancer, leukoplakia, early diagnosis

## Abstract

Oral squamous cell carcinoma (OSCC) is often diagnosed at a late stage and may be malignantly transformed from oral leukoplakia (OL). This study aimed to identify potential plasma microRNAs (miRNAs) for the early detection of oral cancer. Plasma from normal, OL, and OSCC patients were evaluated. Small RNA sequencing was used to screen the differently expressed miRNAs among the groups. Next, these miRNAs were validated with individual samples by quantitative real-time polymerase chain reaction (qRT-PCR) assays in the training phase (*n* = 72) and validation phase (*n* = 178). The possible physiological roles of the identified miRNAs were further investigated using bioinformatics analysis. Three miRNAs (miR-222-3p, miR-150-5p, and miR-423-5p) were identified as differentially expressed among groups; miR-222-3p and miR-423-5p negatively correlated with T stage, lymph node metastasis status, and clinical stage. A high diagnostic accuracy (Area under curve = 0.88) was demonstrated for discriminating OL from OSCC. Bioinformatics analysis reveals that miR-423-5p and miR-222-3p are significantly over-expressed in oral cancer tissues and involved in various cancer pathways. The three-plasma miRNA panel may be useful to monitor malignant progression from OL to OSCC and as potential biomarkers for early detection of oral cancer.

## 1. Introduction

Oral squamous cell carcinoma (OSCC) is the most common type (84–97%) of oral cancer [1]. In South and Southeast Asia and Taiwan, the major risk factor is betel quid chewing. The five-year survival rates for early- and late-stage oral cancer are approximately 82% and 20%, respectively [1]. Unfortunately, around 50% of oral cancer patients present at an advanced stage (TNM III or IV) [2,3], signifying the importance of early diagnosis. Malignant transformation of mucosal lesions predispose to oral cancer. The World Health Organization (WHO) defined these lesions as “potentially malignant disorders (PMD)”. In Taiwan, oral leukoplakia (OL) is the most common oral precancerous lesion [4] and usually transforms into OSCC after five years. Betel quid chewing, alcohol consumption and smoking habits have been indicated to increase the risk of malignant transformation [5,6]. The rate of dysplastic or malignant transformation is between 15.6% and 39.2% [7,8,9]. Thus, the assessment and follow-up of OL should be focused for the early detection of OSCC.

MicroRNAs (miRNAs) are a large family of about 22-nucleotide-long, non-coding, single-stranded RNA molecules that interact with target sequences to degrade or repress translation [9]. They have also been documented to have roles in all of the cancer hallmarks [10] by acting as oncogenes or tumor suppressor genes. Recent studies have revealed the association of miRNA deregulation and its role in OSCC. The most reported possible players in the tumorigenesis of OSCC were miR-21, miR-221, miR-184, miR-133a, miR-375, and let-7b [11]. Besides, over expression of miR-21, miR-181, and miR-345 is associated with the malignant transformation of OL [12]. Three-miRNA signatures (miR-129-5p, miR-339-5p, and miR-31-3p) have been identified as mediators in the initiation and progression of non-malignant to aggressive type of OL [13]. These studies were based on tissue expression, which might not be a practical tool for clinical diagnosis.

Circulating miRNA is an ideal biomarker for the diagnosis and assessment of disease progression and metastasis [14], due to its stability in the extracellular environment and accessibility in various body fluids [15,16]. In previous studies, circulating miRNAs were identified by comparing normal patients to those with OSCC. However, if PMD patients are not included, miRNAs might not help to monitor the transformation from PMD to OSCC, thus missing out on the opportunity to diagnose early OSCC. Moreover, the reference miRNAs for normalization of quantitative real-time polymerase chain reaction (qRT-PCR) data used in past studies, such as RNU-6B, have been variably expressed as in serum and plasma [17]; this might question the reliability of qPCR data.

In the present study, we aimed to identify circulating miRNAs as potential biomarkers for the early detection of OSCC. Moreover, suitable reference miRNA for OL and OSCC populations were also investigated. We collected plasma from normal, OL, and OSCC patients and designed a three phase study to investigate potential biomarkers. In the screening phase, miRNA expression was profiled by small RNA sequencing platform in order to identify differentially expressed miRNAs and reference miRNAs. Subsequently, we used qRT-PCR assays to confirm miRNA expression in individual samples and refined the number of miRNAs. Potential miRNA biomarkers were accessed by an independent cohort in the validation phase. Our results provided a three-miRNA panel for discrimination between normal, OL, and OSCC patients.

## 2. Results

### 2.1. Characteristics of Study Subjects

We recruited 250 participants (including 72 and 178 participants in training and validation phases, respectively) (Table 1). All of the OSCC patients were free from distant metastasis. There was no age or gender distribution difference between the screening/training phase (*p* = 0.960, 0.453, respectively) and validation phase (*p* = 0.130, 0.877, respectively).

### 2.2. Expression Profiling of miRNAs by Small RNA-seq

**Figure 1:**
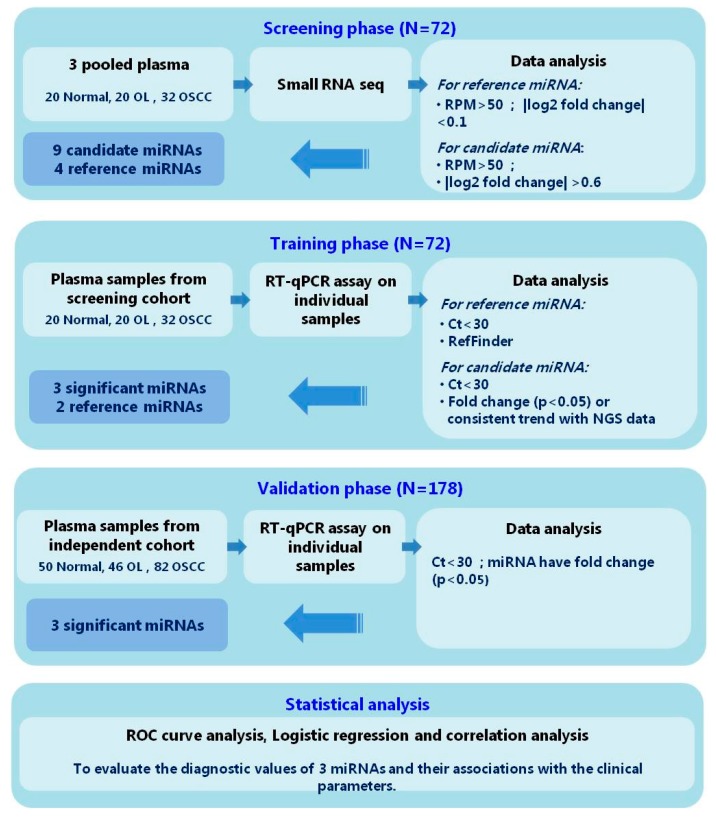
Study design. Abbreviations: RPM = reads per million; qRT-PCR = quantitative reverse transcription polymerase chain reaction; ROC = receiver operating characteristics.

**Figure 2:**
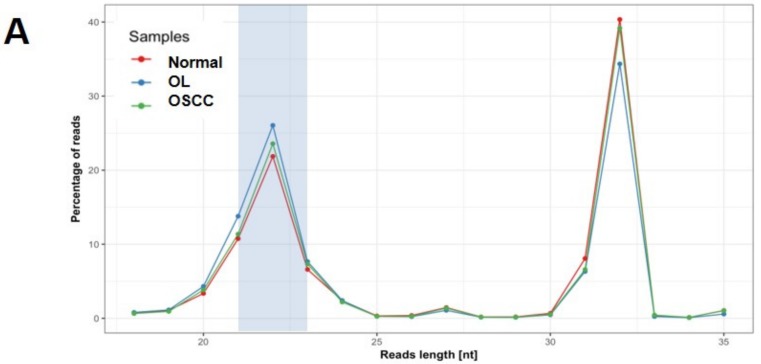

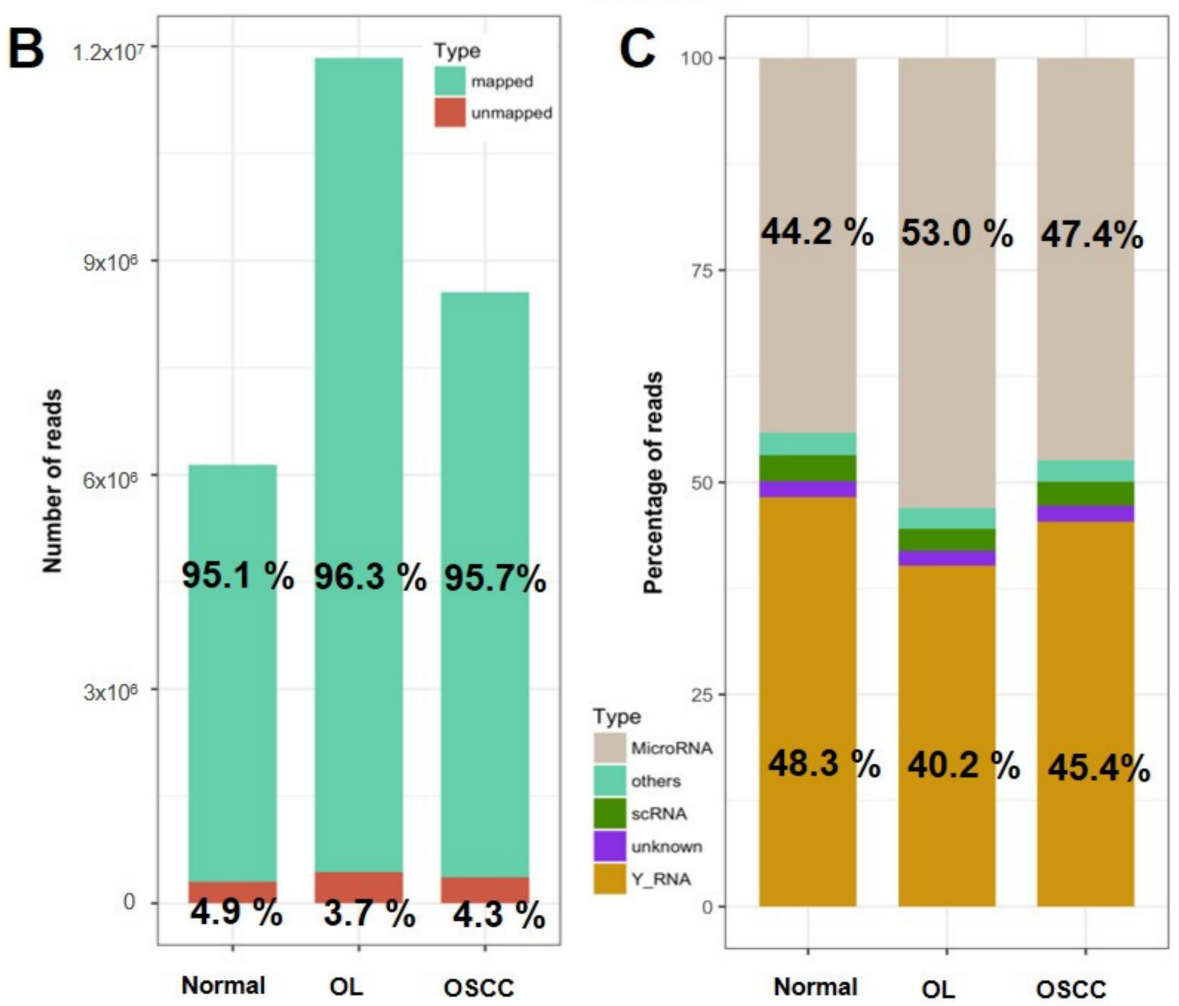
Quality of small RNA sequencing data. (**A**) Read length distribution indicates that all the samples with a peak in read length 21–23 of miRNA length. (**B**) More than 95% reads were mapped to reference genome. (**C**) More than 44% of mapped reads were miRNAs.

**Figure 3:**
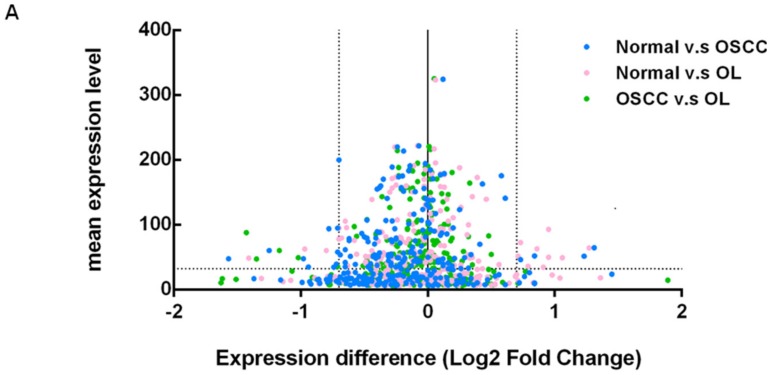

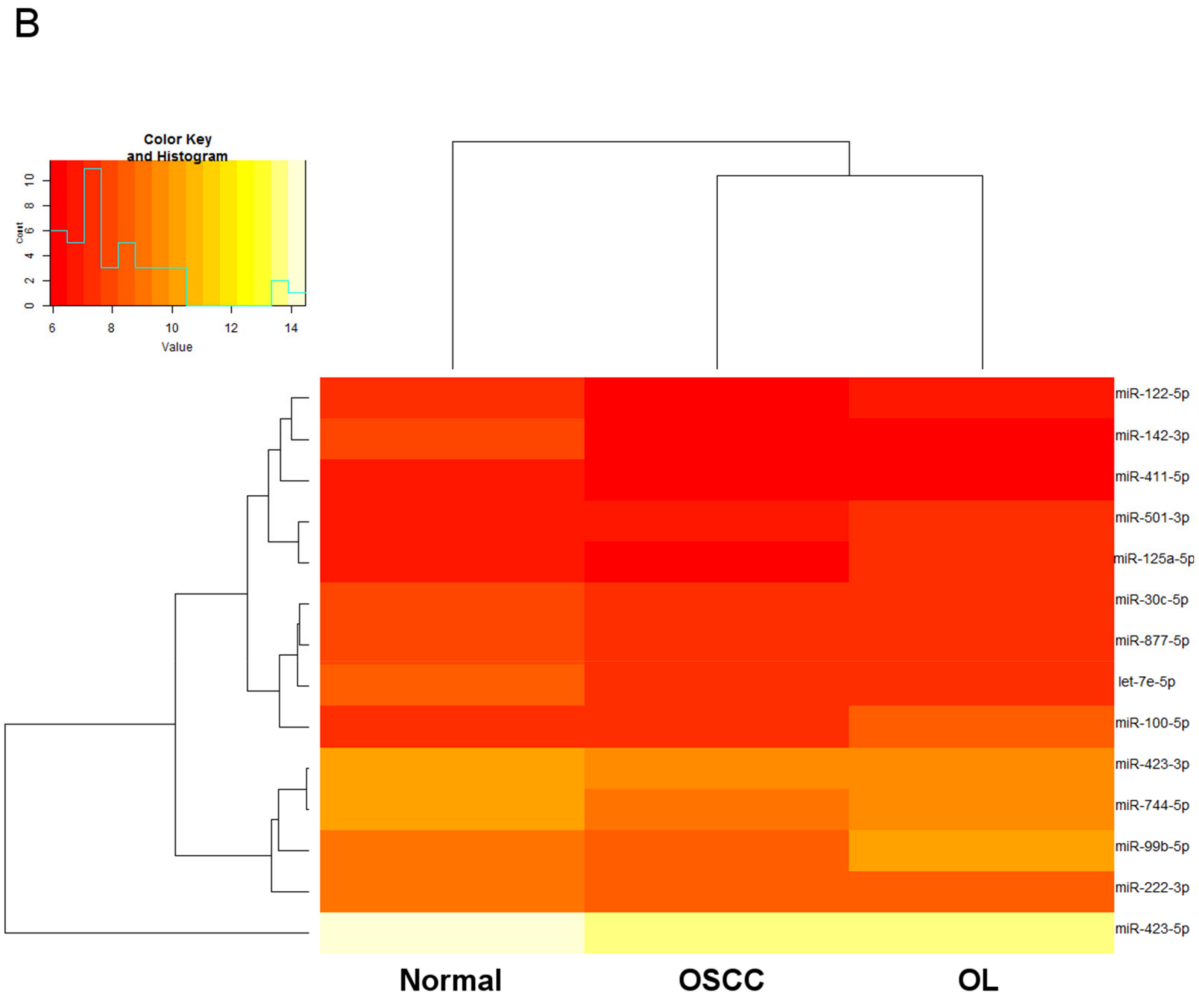
miRNA profiling by small RNA sequencing. (**A**) Each point indicates the expression difference of a single miRNA between specified groups. The dotted line represents the cut-off value of expression difference and expression level while the solid line indicates no discrepant expression among groups. (**B**) Fourteen deregulated miRNAs. The miRNA expression level (log2 transformed RPM value) is presented. The color legend from red to white means the expression value from 6 to 14, and the light blue line presents a histogram of the expression value.

As illustrated in Figure 1, small RNA-seq analysis was performed on three pooled samples to identify the differentially expressed miRNA. NGS raw data was uploaded and submitted to a public repository Gene Expression Omnibus (GEO) database (GSE104440). Small RNA reads were highly qualified (Figure 2) for subsequent analysis. Differentially expressed miRNA (Figure 3A) were identified according to the criteria detailed in Figure 1. Total of 14 miRNAs (Figure 3B) were found to be deregulated between normal/OL, OL/OSCC, or OSCC/normal groups. Among the three sets of comparisons, nine candidate miRNA were deregulated in any two sets and were selected for subsequent confirmation.

We tried to identify potential reference miRNA across normal, OL, and OSCC patients for relative quantification by qRT-PCR. Potential reference miRNA were identified if they met the criteria, as illustrated in Figure 1. Four miRNA (miR-130b-3p, miR-221-3p, miR-101-3p, and miR-16-5p) were selected for further analysis.

### 2.3. Selection of Suitable Reference miRNAs

The expression levels of four reference miRNAs were confirmed by qRT-PCR in 72 individual samples (Figure 1). Four miRNAs were detected among 72 samples with median *C*_t_ <30 and were considered suitable. By analyzing the *C*_t_ values, miR-101-3p revealed differential expression among groups (*p* = 0.001) and was excluded (Supplementary Table S1). Stability was investigated by RefFinder, which is an online tool integrating four programs (http://150.216.56.64/referencegene.php); lower values indicate greater stability. As a result, miR-130b-3p and miR-221-3p were combined and selected as the reference miRNA set (Supplementary Table S1).

### 2.4. Investigation of the Six Candidate miRNAs in Training Phase

Three out of nine candidate miRNAs were excluded, owing to the relatively low expression levels (*C*_t_ > 30) in pooled samples. The remaining six miRNAs (miR-let-7e-5p, miR-222-3p, miR-423-5p, miR-150-5p, miR-125a-5p, and miR-100-5p) were investigated in individual samples.

**Figure 4:**
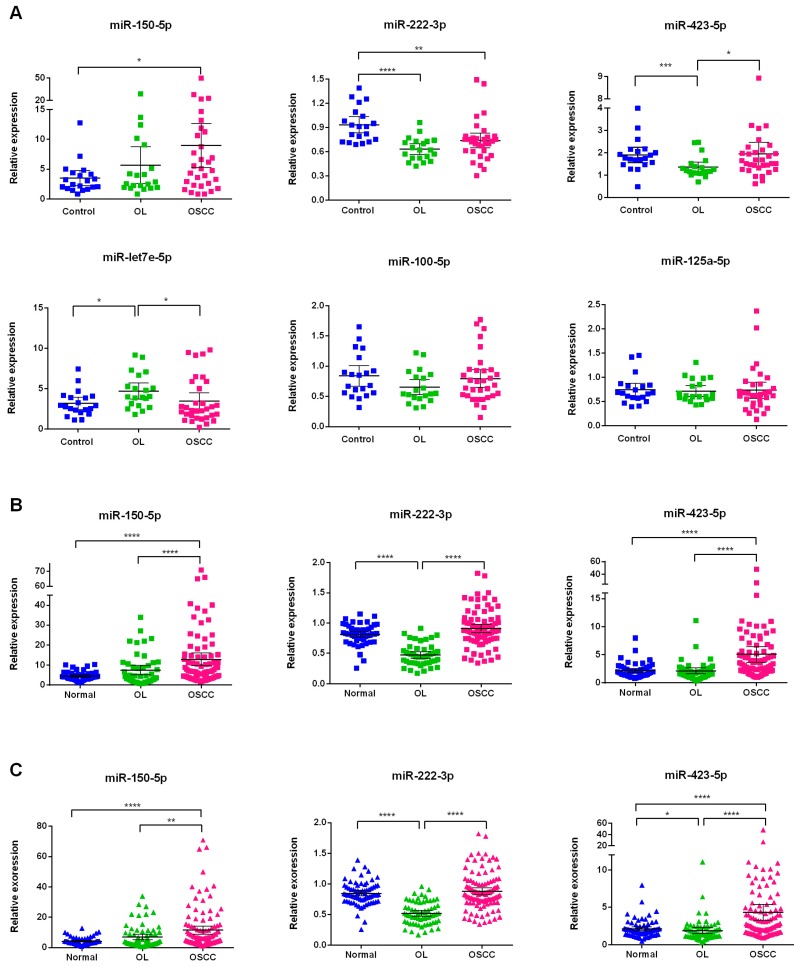
Expression of candidate miRNA in different data sets. (**A**) Abundance of miR-let-7e-5p, miR-125a-5p, miR-100-5p, miR-150-5p, miR-222-3p, and miR-423-5p in plasma of subjects in training phase (*n* = 72). Expression of significant miRNA (miR-222-3p, miR-423-5p, and miR-150-5p) in plasma from validation phase (*n* = 178) (**B**) and from all subjects (*n* = 250) (**C**). Significance of two-sided *p*-values is indicated as follows: * *p* < 0.05, ** *p* < 0.01, *** *p* < 0.001, and **** *p* < 0.001 (Mann–Whitney test).

The relative expression level of each miRNA among groups was obtained by normalization with reference miRNA set (mean *C*_t_) by comparative *C*_t_ method. Significant miRNAs were chosen according to the criteria listed in Figure 1. No significantly different miRNA levels of miR-125a-5p and miR-100-5p were observed (Figure 4A) between groups. In addition, miR-let-7e-5p displayed an inconsistent trend with NGS profiling data (Supplementary Figure S1). Taken together, three miRNAs (miR-222-3p, miR-423-5p, and miR-150-5p) were considered significant for the next validation.

### 2.5. Validation of Three Significant miRNA with an Independent Cohort

An independent cohort was used to validate the expression levels of the three miRNAs (Figure 1). We found that miR-222-3p was found to be significantly down regulated in OL patients when compared to that in normal and OSCC patients (*p* < 0.0001), whereas miR-423-5p and miR-150-5p increased in OSCC patients as compared to those in normal and OL patients (*p* < 0.001, Figure 4B). Similar results were observed when analyzing all of the samples (Figure 4C) from training and validation phases. These results reveal that the three miRNA could be potential biomarkers for the diagnosis of OL and OSCC.

### 2.6. Correlations between miRNA Signature and Clinical Parameters

**Figure 5:**
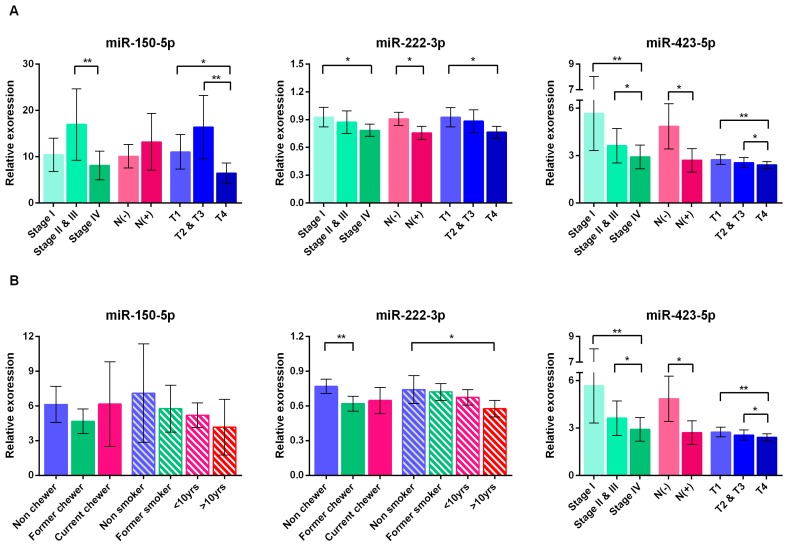
Expression of miR-222-3p, miR-423-5p, and miR-150-5p in different groups of patients. (**A**) OSCC patients (*n* = 114) were categorized according to clinical stage, lymph node metastasis status indicates the presence [N(+)] and absence [N(−)] of metastasis and T stage. (**B**) Normal and oral leukoplakia (OL) patients (*n* = 136) were classified by the habit of smoking and betel nut chewing. Current smokers were further divided into two groups according to the history of smoking. Significance of two-sided *p*-values is indicated as follows: * *p* < 0.05, ** *p* < 0.01 (Mann–Whitney test).

Spearman rank analysis showed that miR-222-3p and miR-423-5p negatively correlated with clinical stage, lymph node metastasis status, and T stage (Table A1). For OSCC patients, miR-222-3p, and miR-423-5p significantly down regulated when tumors spread to lymph node (*p* = 0.026, 0.019, respectively), and gradually declined with tumor progression (Figure 5A). Although miR-150-5p did not correlate with the node metastasis and tumor progression, decreased the expression level at late-stage tumor was observed (Figure 5A). These findings imply that miR-222 and miR-423-5p could be predictors for tumor progression.

In non-cancer patients, betel quid chewing was shown to have negative correlation with miR-222-3p level (Table A1). A significant difference was observed in miR-222-3p level between non-chewers and former chewers (*p* = 0.001); long-term smokers (>10 years); and, non-smokers (*p* = 0.032) (Figure 5B). However, the miRNA abundance neither correlated with (Table A1) nor showed difference among patients with different drinking habits.

### 2.7. Logistic Regression Analysis of miRNA Biomarkers

For all of the patients, regression analysis was conducted to determine the diagnostic efficacy of three miRNA signatures. In model 1, we set the normal group as the reference category. The relative risks (RRs) of these miRNAs for OL were shown in Table 2. These results reveal that miR-222-3p and miR-150-5p were independently associated with OL, whereas miR-150-5p and miR-423-5p associated with OSCC. Similarly, we found that all three miRNAs were significant independent predictors of OSCC when the OL group was treated as the reference category (Table 2). In addition, multivariate logistic regression analysis adjusted for sex, age, smoking, and betel quid chewing habits were also performed. As with univariate logistic regression analysis, these independent associations still remained significant (Table 2).

Furthermore, logistic regression analysis was used to conduct a risk score analysis to identify the best combinations of miRNAs to predict OL and OSCC. The combination of miR-222-3p and miR-150-5p, miR-150-5p, and miR-423-5p, produced the best model to predict OL and OSCC, respectively. A combination of the three miRNAs enabled distinction between OL and OSCC (Table 2).

### 2.8. Diagnostic Performance of miRNA Signature

**Figure 6:**
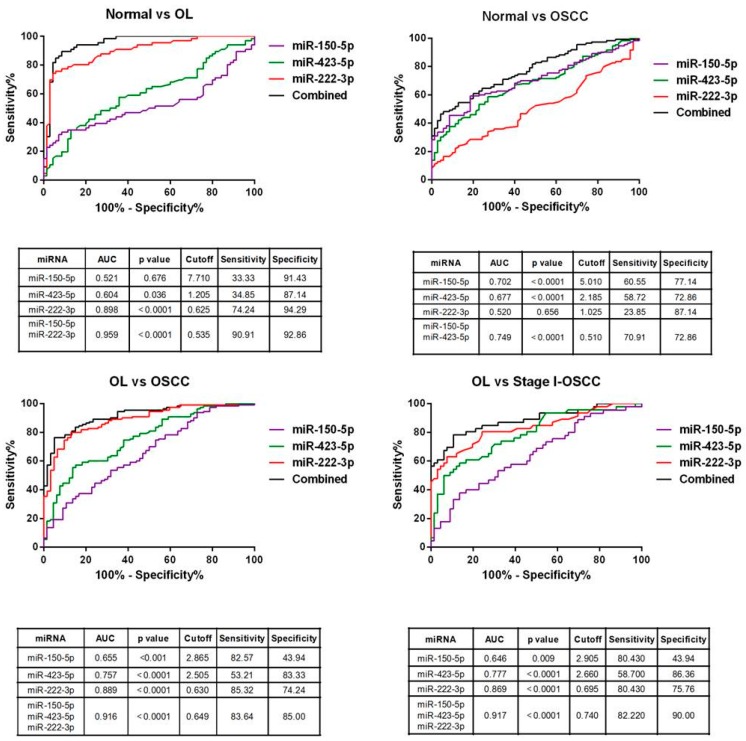
ROC analysis for individual miRNA and combined panel. ROC curve was generated by analyzing samples from all patients. The combined panels were generated by linear combination of values of each miRNA. AUC = area under the ROC curve. *p*-Values were calculated using Mann–Whitney test.

Receiver operating characteristic (ROC) analysis was applied on the miRNAs in all of the subjects (Figure 6) after being adjusted by the multivariate model. We also evaluated the diagnostic value of the combined miRNA panel. The combination of miRNAs panel increased the AUC when individually when compared with any of the miRNAs (Figure 6). The area under curve (AUC) for combined miRNA panel was 0.959 (miR-150-5p/miR-222-3p, 95% CI, 0.927–0.991, *p* < 0.0001) and 0.749 (miR-150-5p/miR-423-5p, 95% CI, 0.678–0.819, *p* < 0.0001) for OL and OSCC patients, respectively. The three miRNA combined panel for distinguishing OL from OSCC patients yielded an AUC value of 0.916 (95% CI, 0.874–0.957, *p* < 0.0001).

We observed lower miRNA expression at different stages of OSCC; therefore, we considered whether these miRNAs may discriminate between OL and early (stage I) OSCC. The three-miRNA panel yielded an AUC value of 0.917 (95% CI, 0.861–0.973, *p* < 0.0001) (Figure 6). Accordingly, these data suggest that different combinations of the three miRNAs serve as potential biomarkers for OL and OSCC. Importantly, the three-miRNA panel helped detection of transformation from OL to early malignancy.

### 2.9. Bioinformatics Analysis of miR-222-3p, miR-423-5p, and miR-150-5p

Circulating miRNA could originate from tumor cells. To elucidate this, we analyzed the miRNA expression profiles of solid tissue of head and neck cancer from The Cancer Genome Atlas (TCGA, http://cancergenome.nih.gov/). Tissue miR-222-3p and miR-423-5p levels were significantly up-regulated in OSCC patients (Supplementary Figure S2) when compared to those in normal (*p* < 0.0001). Furthermore, tissue miR-222-3p was down regulated in OSCC with lymph node metastasis, similar to the levels observed in OSCC plasma. Although tissue miR-150-5p was not found to be differently expressed between normal and OSCC patients, a gradual decrease was observed in miR-150-5p with tumor growth (Supplementary Figure S2).

Putative predicted target genes of miRNA and experimentally validated miRNA-gene interactions were included for pathway enrichment analysis. The identified pathways (Table 3) reveal the involvement of these miRNAs in cancer-related pathways, such as Wnt, PI3K-Akt, MAPK, and Ras signaling pathway. Among these pathways, Wnt signaling pathway, significant in head and neck cancer, was the most enriched pathway. IPA analysis also indicated that Wnt signaling was the one of the most enriched pathways (Supplementary Figure S3) and cancer was the top enriched disease (Supplementary Table S2). These results provided a clue of the roles played by these miRNAs in OSCC.

## 3. Discussion

To date, histopathology remains as the golden standard for reporting cancer risk of PMD. The invasiveness of histopathology leads to poor compliance for patients and is impossible to be used for monitoring the disease progression. Thus, non-invasive tools, such as tolonium chloride or toluidine blue dye, Oral CDx brush biopsy and latest optical systems (e.g., Vizilite and Velscope) were developed to detect precancer lesions [18]. Unfortunately, morphological finding only indicates the malignant potential (dysplasia) of a given lesion at that time, whereas subtle molecular changes can be detected before the morphological changes. Therefore, molecular biomarkers for detection of oral cancers have been developed and extended to point of care tests (e.g., IL-8 and IL-8 mRNA) [19,20]. 

This study aims to identify plasma miRNAs as biomarkers for early detection of OSCC. We found that miR-130b-3p and miR-221-3p were the most suitable reference miRNAs in this population. The expression levels of three miRNAs, miR-222-3p, miR-150-5p, and miR-423-3p, were found to be different between groups. For non-cancer patients, miR-222-3p correlated with betel chewing, whereas miR-222-3p and miR-423-5p were associated with tumor progression and lymph node metastasis. Among these miRNAs, the combination of miR-150-5p/miR-222-3p and miR-150-5p/miR-423-5p best discriminated normal from OL and OSCC, respectively. Importantly, we demonstrated that a three-miRNA panel can be used in OL patients for early detection of OSCC. 

Several studies demonstrated the differential miRNA levels between the plasma of OSCC and normal [21,22,23]; Yang et al., 2011a [24]. However, little is known about circulating miRNA in OL. Only one study [25] revealed that salivary miR-31 was lower in OL compared to that in OSCC. The miRNA alteration we observed in this study differs from that in previous reports. Recent studies have indicated some pre-analytical and analytical factors causing these problems, e.g., sample type, extraction methods, and measurement platforms [26,27]. Most studies used RNU-6 and miR-16 as reference genes for the relative quantification of target miRNA. Unfortunately, RNU-6 unstably expressed in plasma and serum [17], whereas miR-16 was affected by hemolysis [28]. Instead of using RNU-6 and miR-16, we identified miR-221-3p and miR-130b-3p as suitable miRNA for our study. These factors might contribute to the different findings we observed. 

In the screening phase, we used a small RNA sequencing platform than using microarray, which is widely used in previous studies. With this technique, it is possible to profile miRNA without knowing the sequence of miRNA beforehand and is powerful for miRNA discovery [29]. The application of NGS to measure miRNAs in serum/plasma is still in its early phase. To our knowledge, there was only one study utilized NGS strategy to profile plasma miRNA in the field of oral oncology and identified plasma miRNA biomarkers to monitor OSCC recurrence in patients after surgery [30]. 

It was indicated that miR-222 was co-transcribed in a cluster with miR-221, and the expression of these two miRNAs were shown to be highly correlated in OSCC [31]. Most recently, down regulation of miR-221/222 was shown to promote apoptosis in OSCC cells [32]. The expression levels of reference miRNA sets, miR-130b, and miR-221-3p, were used to calculate the relative expression level of miR-222-3p in this study. Therefore, not only the correlations between miR-221-3p and miR-222-3p but also the independence of the reference miRNA sets were examined. We found that the reference miRNA sets were not correlated with any of the three identified miRNAs. Moreover, the high correlated expression level between miR-221 and miR-222 demonstrated in previous tissue or cell line studies were not observed in the present study (ρ = −0.221, *p* = 0.061).

The limitation of this study is the limited source of tissue samples, especially for OL and early stage OSCC. Therefore, we investigated the tissue expression levels of the three identified miRNAs by analyzing the HNSC data set from TCGA. Among them, miR-222-3p was confirmed to have higher expression level in OSCC. A previous study indicated increased miR-222 expression that was found in 40% OSCC and was correlated with tumor growth [31]. We found that tissue and plasma miR-222-3p was down regulated in OSCC if lymph node metastasis were present. A previous study also demonstrated that miR-222-3p contributed to metastasis in tongue cancer by targeting matrix metalloproteinase 1 and manganese superoxide dismutase 2. Ectopic transfection of miR-222-3p resulted in aberrant decrease in cell invasion and migration [33]. Other studies suggested that miR-222 affected cell growth, invasive and apoptotic abilities by targeting to PUMA in OSCC [34,35]. Our analysis also revealed that tissue miR-150-5p declined with tumor progression. The expression of vascular endothelial growth factor A, which is the target gene of miR-150-5p [36], was significantly associated with the tumor stage [37]. In OSCC, increased expression of miR-423-5p was demonstrated in plasma and tissues; however, another study reported a down-regulation in miR-423 in OSCC tissues [38]. Nonetheless, these findings suggest that the identified miRNA in this study acts as an oncomiR during tumor development.

Among the three identified miRNAs, we observed the expressions of miR-222-3p and miR-150-5p did not match the results from TCGA analysis. Up-regulation of miR-222-3p in OSCC was found in tissue but not in plasma when compared to normal. On the other hand, the up-regulation of miR-150-5p in OSCC was only found in plasma. Regarding the consistency of expression level between circulating miRNA and tissue miRNA, only a limited number of studies addressed this issue. Findings were controversial: some researchers described a similar trend of alteration, both in circulating and tissue miRNAs [39,40], whereas others observed the inconsistency between cellular miRNA and circulating miRNA [41,42,43,44]. Moreover, Pigati et al. [45] suggested the existence of a cellular selection mechanism for miRNA release and indicated that the extracellular and cellular miRNA profiles differ.

Pathway enrichment analysis also revealed possible functions of the identified miRNAs. Our results suggest that Wnt signaling pathway was the most enriched pathway. The deregulation of Wnt signaling pathway promoted the development and progression of oral cancer; it is also associated with prognosis in OSCC. Of note, β-catenin, which is a downstream mediator of Wnt signaling pathway, was demonstrated to be involved in oral malignant transformation. In dysplastic oral tissues or cancer tissues, β-catenin translocated form membrane to cytoplasm or nucleus [46]. A recent study revealed up-regulation in MAPK, ERK, JNK, IL-6/STAT3, WNT, TGFβ, and glucocorticoid receptor signaling to be the possible driving force behind the early stages of OSCC tumorigenesis [47]. Taken together, our results suggest that the three identified miRNAs might play important roles in the early stage OSCC development. However, the underlying mechanisms are beyond the scope of this study and will be further investigated in the future.

In conclusion, we identified three plasma miRNA for the detection of OL and OSCC by integration of small RNA sequencing and qRT-PCR platforms. By different combination of these three miRNA, OL and OSCC could be diagnosed. The three-miRNA panel demonstrated a high diagnostic value for discriminating OL from OSCC, and could be useful in the follow-up of OL and early detection of OSCC. Our results provide the basis of applying circulating miRNA to monitor malignant transformation of OL and could be extended to other PMD to benefit OSCC patients.

## 4. Materials and Methods 

### 4.1. Clinical Samples

Two hundred and fifty patients (70 normal, 66 OL, and 114 OSCC) at the Chung Shan Medical University Hospital, Taiwan, were recruited between 2013 and 2016. Ethics approval for this study was obtained from the Institutional Review Board of Chung Shan Medical University Hospital (CSMUH No.: CS13214-1, 28 November 2014). Patients’ blood was drawn within two weeks after the diagnosis was confirmed. The whole blood samples were collected in EDTA tubes from each patient after obtaining written informed consent. Plasma was separated by centrifuged at 3000× *g* within two hours after the blood was drawn. RNA extraction from clinical samples was described in the Supplementary Materials and Methods.

### 4.2. Small RNA Library Preparation and Sequencing

Library was constructed by TruSeq Small RNA Preparation Kits (Illumina Inc., San Diego, CA, USA), according to the manufacturer’s instructions. Library was size selected by 6% TBE PAGE gels to remove excess adapter dimmers. The final library size was confirmed by Agilent tape station 2200 (HSD1000 assay). Subsequently, indexed libraries were quantified by KAPA Library Quantification Kit (Kapa Biosystems, Wilmington, MA, USA), and 2 nM of library sample was subjected to NextSeq 500 (Illumina) for cluster generation and sequencing.

### 4.3. Small RNA Sequencing Analysis

Reads of small RNA sequencing were trimmed and processed before mapping to human genome. Detailed information is provided in the Supplementary Materials and Methods.

### 4.4. miRNA Quantification by qRT-PCR Assays

Plasma miRNA was reverse transcribed using miScript II RT Kit (Qiagen) according to the manufacturer’s manual. Subsequent qPCR quantification was performed using miScript SYBR Green PCR Kit (Qiagen) on Rotor-Gene Q (Qiagen) instrument. Each sample was analyzed in triplicate. The *C. elegans* synthetic mir-39 spike-in control was used to normalize and evaluate technical variation in RNA extraction experiment as previously described [48].

### 4.5. Statistical Analysis

Differences in clinical characteristics among patients were compared using χ^2^ test and one-way ANOVA for categorical and continuous variables, respectively. The Mann–Whitney U test was used to compare different miRNAs levels between groups, and data were presented as means ± 95% confidence interval (CI). Spearman rank correlation test was used to determine the association between miRNAs and clinical parameters. ROC curves of individual miRNAs were constructed to obtain the optimal cutoff for the detection of OL and OSCC. The risk score analysis is described in Supplementary Materials and Methods. Statistical analysis was performed using the GraphPad Prism 6 (GraphPad Software, Inc., La Jolla, CA, USA) or SPSS software version 22.0, (SPSS Inc., Chicago, IL, USA). A *p*-value < 0.05 was considered statistically significant.

### 4.6. Bioinformatics Analysis

The miRNA expression profiles of solid tissue from head and neck cancer were analyzed and compared to those of plasma in our observations. In addition, pathway enrichment analysis was conducted to discover potential functional roles of identified miRNAs. Further information is provided in the Supplementary Materials and Methods.

## Figures and Tables

**Table 1:** Clinical characteristics of study subjects.

	Screening and Training Phase (*n* = 72)			Validation Phase (*n* = 178)		
Variables	Normal (%)	OL (%)	OSCC (%)	Normal (%)	OL (%)	OSCC (%)
Number	20	20	32	50	46	82
Age (mean ± SD)	52.05 ± 12.78	52.20 ± 12.53	52.20 ± 9.03	52.86 ± 14.06	48.35 ± 12.11	53.79 ± 11.25
Sex						
Male	20 (100.0)	18 (90.0)	31 (96.8)	48 (96.0)	44 (95.6)	80 (97.5)
Female	0 (0.0)	2 (10.0)	1 (3.2)	2 (4.0)	2 (4.4)	2 (2.5)
Smoking						
Non-smoker	4 (20.0)	6 (30.0)	1 (3.2)	2 (4.0)	6 (13.0)	7 (8.5)
Former smoker	9 (45.0)	11 (55.0)	12 (37.5)	15 (30.0)	12 (26.1)	16 (19.5)
Current smoker	7 (35.0)	3 (15.0)	19 (59.3)	33 (66.0)	28 (60.9)	59 (72.0)
BQ chewing						
Non-BQ	11 (55.0)	6 (30.0)	3 (9.4)	27 (54.0)	12 (26.1)	7 (8.5)
Former BQ chewing	6 (30.0)	10 (50.0)	25 (78.1)	15 (30.0)	28 (60.9)	63 (76.8)
Current BQ-chewing	3 (15.0)	4 (20.0)	4 (12.5)	8 (16.0)	6 (13.0)	12 (14.7)
Alcohol consumption						
Non-drinker	5 (25.0)	9 (45.0)	11 (34.4)	20 (40.0)	20 (43.5)	22 (26.8)
Former drinker	10 (50.0)	9 (45.0)	13 (40.6)	22 (44.0)	23 (50.0)	36 (43.9)
Current drinker	5 (25.0)	2 (10.0)	8 (25.0)	8 (16.0)	3 (6.5)	24 (29.3)
Stage						
I			14 (43.8)			32 (39.0)
II			0 (0.0)			15 (18.3)
III			0 (0.0)			11 (13.4)
IV			18 (56.2)			24 (29.3)
T stage						
T1			14 (43.8)			33 (40.2)
T2			3 (9.4)			24 (29.3)
T3			0 (0.0)			4 (4.9)
T4			15 (46.8)			21 (25.6)
N stage						
N0			19 (59.4)			60 (73.2)
N1			5 (15.6)			11 (13.4)
N2			8 (25.0)			11 (13.4)

Abbreviations: OL = oral leukoplakia; OSCC = oral squamous cell carcinoma; BQ = betel quid; SD = standard deviation.

**Table 2:** Regression analysis.

	Univariate		Mulitivariate	
	Relative Risk	*p*-Value	Relative Risk	*p*-Value
Model 1				
OL				
miR-222-3p	0.205 (0.123−0.344)	<0.001	0.212 (0.127−0.357)	<0.001
miR-150-5p	1.114 (1.027−1.210)	0.010	1.124 (1.032−1.223)	0.007
miR-423-5p	0.880 (0.673−1.150)	0.349	0.897 (0.682−1.180)	0.437
miR panel ^a^	1.348 (1.233−1.474)	<0.001	1.361 (1.238−1.496)	<0.001
OSCC				
miR-222-3p	1.038 (0.858−1.256)	0.699	1.114 (0.898−1.383)	0.324
miR-150-5p	1.189 (1.083−1.306)	<0.001	1.198 (1.079−1.330)	0.001
miR-423-5p	1.466 (1.182−1.817)	<0.001	1.599 (1.238−2.066)	<0.001
miR panel ^b^	1.377 (1.198−1.584)	<0.001	1.386 (1.189−1.615)	<0.001
Model 2				
OSCC				
miR-222-3p	2.915 (2.087−4.072)	<0.001	3.014 (2.102−4.321)	<0.001
miR-150-5p	1.038 (1.003−1.075)	0.035	1.048 (1.007−1.091)	0.020
miR-423-5p	1.581 (1.238−2.021)	<0.001	1.601 (1.236−2.075)	<0.001
miR panel ^c^	1.455 (1.308−1.617)	<0.001	1.448 (1.292−1.623)	<0.001

The reference category was normal and OL patients in mode 1 and model 2, respectively. ^a^ miR222-3p and miR-150-5p; ^b^ miR-150-5p and miR-423-5p; ^c^ miR-222-3p, miR-150-5p, and miR-423-5p.

**Table 3:** The top 10 most enriched KEGG pathway with the targetome of three miRNAs.

Term	No. of Genes	*p*-Value	Fold Enrichment
Wnt signaling pathway	16	4.69 × 10^−6^	4.22
Pathways in cancer	26	6.07 × 10^−5^	2.41
Hepatitis B	14	1.55 × 10^−4^	3.51
Axon guidance	13	1.70 × 10^−4^	3.72
Sphingolipid signaling pathway	11	1.58 × 10^−3^	3.33
HTLV-I infection	17	1.66 × 10^−3^	2.42
PI3K-Akt signaling pathway	20	2.71 × 10^−3^	2.11
Proteoglycans in cancer	14	3.19 × 10^−3^	2.55
FoxO signaling pathway	11	3.57 × 10^−3^	2.99
Rap1 signaling pathway	14	4.79 × 10^−3^	2.42
MicroRNAs in cancer	17	4.84 × 10^−3^	2.17

KEGG: Kyoto Encyclopedia of Genes and Genomes.

## Supplementary Materials

Supplementary materials can be found at http://www.mdpi.com/1422-0067/19/3/758/s1.

## Abbreviations

OSCC	Oral squamous cell carcinoma
OL	Oral leukoplakia
miRNAs	MicroRNAs
qRT-PCR	Quantitative real-time polymerase chain reaction
PMD	Potentially malignant disorders
ROC	Receiver operating characteristic
AUC	Area under curve
TCGA	The Cancer Genome Atlas

## Appendix A

**Table A1:** Spearman correlation analysis.

	mir-222-3p		mir-423-5p		mir-150-5p	
Variables	ρ	*p*-Value	ρ	*p*-Value	ρ	*p*-Value
Age	0.105	NS	0.037	NS	−0.052	NS
Gender	0.039	NS	0.004	NS	0.103	NS
Betel chewing status	−0.241	0.005	−0.149	NS	−0.099	NS
Smoking status	−0.161	NS	0.034	NS	0.001	NS
Alcohol status	−0.121	NS	0.086	NS	−0.178	NS
Clinical stage	−0.201	0.032	−0.237	0.011	−0.116	NS
T stage	−0.220	NS	−0.276	0.003	−0.156	NS
Lymph node metastasis	−0.222	0.018	−0.220	0.019	0.012	NS

NS: Not significant.

## References

[1] Corso G.D., Villa A., Tarsitano A., Gohel A. (2016). Current trends in oral cancer: A systematic review. Cancer Cell Microenviron..

[2] Lo W.L., Kao S.Y., Chi L.Y., Wong Y.K., Chang R.C. (2003). Outcomes of oral squamous cell carcinoma in Taiwan after surgical therapy: Factors affecting survival. J. Oral Maxillofac. Surg..

[3] Chen Y.K., Huang H.C., Lin L.M., Lin C.C. (1999). Primary oral squamous cell carcinoma: An analysis of 703 cases in southern Taiwan. Oral Oncol..

[4] Kao S.Y., Chu Y.W., Chen Y.W., Chang K.W., Liu T.Y. (2009). Detection and screening of oral cancer and pre-cancerous lesions. J. Chin. Med. Assoc..

[5] Jeng J.H., Chang M.C., Hahn L.J. (2001). Role of areca nut in betel quid-associated chemical carcinogenesis: Current awareness and future perspectives. Oral Oncol..

[6] Yeh C.Y., Lin C.L., Chang M.C., Chen H.M., Kok S.H., Chang S.H., Kuo Y.S., Hahn L.J., Chan C.P., Lee J.J. (2016). Differences in oral habit and lymphocyte subpopulation affect malignant transformation of patients with oral precancer. J. Formos. Med. Assoc..

[7] Neville B.W., Day T.A. (2002). Oral cancer and precancerous lesions. CA Cancer J. Clin..

[8] Mortazavi H., Baharvand M., Mehdipour M. (2014). Oral potentially malignant disorders: An overview of more than 20 entities. J. Dent Res. Dent Clin. Dent Prospect..

[9] Zhang B., Pan X., Cobb G.P., Anderson T.A. (2007). microRNAs as oncogenes and tumor suppressors. Dev. Biol..

[10] Hanahan D., Weinberg R.A. (2011). Hallmarks of cancer: The next generation. Cell.

[11] Gorenchtein M., Poh C.F., Saini R., Garnis C. (2012). MicroRNAs in an oral cancer context—from basic biology to clinical utility. J. Dent. Res..

[12] Cervigne N.K., Reis P.P., Machado J., Sadikovic B., Bradley G., Galloni N.N., Pintilie M., Jurisica I., Perez-Ordonez B., Gilbert R. (2009). Identification of a microRNA signature associated with progression of leukoplakia to oral carcinoma. Hum. Mol. Genet..

[13] Maimaiti A., Abudoukeremu K., Tie L., Pan Y., Li X. (2015). MicroRNA expression profiling and functional annotation analysis of their targets associated with the malignant transformation of oral leukoplakia. Gene.

[14] Krysan K., Kusko R., Grogan T., O’Hearn J., Reckamp K.L., Walser T.C., Garon E.B., Lenburg M.E., Sharma S., Spira A.E. (2014). PGE2-driven expression of c-Myc and oncomiR-17-92 contributes to apoptosis resistance in NSCLC. Mol. Cancer Res..

[15] Mitchell P.S., Parkin R.K., Kroh E.M., Fritz B.R., Wyman S.K., Pogosova-Agadjanyan E.L., Peterson A., Noteboom J., O’Briant K.C., Allen A. (2008). Circulating microRNAs as stable blood-based markers for cancer detection. Proc. Natl. Acad. Sci. USA.

[16] Ng E.K., Chong W.W., Jin H., Lam E.K., Shin V.Y., Yu J., Poon T.C., Ng S.S., Sung J.J. (2009). Differential expression of microRNAs in plasma of patients with colorectal cancer: A potential marker for colorectal cancer screening. Gut.

[17] Wang K., Yuan Y., Cho J.H., McClarty S., Baxter D., Galas D.J. (2012). Comparing the MicroRNA spectrum between serum and plasma. PLoS ONE.

[18] Messadi D.V. (2013). Diagnostic aids for detection of oral precancerous conditions. Int. J. Oral Sci..

[19] Khan R.S., Khurshid Z., Asiri F.Y.I. (2017). Advancing Point-of-Care (PoC) Testing Using Human Saliva as Liquid Biopsy. Diagnostics.

[20] Torrente-Rodriguez R.M., Campuzano S., Ruiz-Valdepenas Montiel V., Gamella M., Pingarron J.M. (2016). Electrochemical bioplatforms for the simultaneous determination of interleukin (IL)-8 mRNA and IL-8 protein oral cancer biomarkers in raw saliva. Biosens. Bioelectron..

[21] Lu Y.C., Chang J.T., Huang Y.C., Huang C.C., Chen W.H., Lee L.Y., Huang B.S., Chen Y.J., Li H.F., Cheng A.J. (2015). Combined determination of circulating miR-196a and miR-196b levels produces high sensitivity and specificity for early detection of oral cancer. Clin. Biochem..

[22] Liu C.J., Tsai M.M., Tu H.F., Lui M.T., Cheng H.W., Lin S.C. (2013). miR-196a overexpression and miR-196a2 gene polymorphism are prognostic predictors of oral carcinomas. Ann. Surg. Oncol..

[23] Hung P.S., Liu C.J., Chou C.S., Kao S.Y., Yang C.C., Chang K.W., Chiu T.H., Lin S.C. (2013). miR-146a enhances the oncogenicity of oral carcinoma by concomitant targeting of the IRAK1, TRAF6 and NUMB genes. PLoS ONE.

[24] Liu C.J., Kao S.Y., Tu H.F., Tsai M.M., Chang K.W., Lin S.C. (2010). Increase of microRNA miR-31 level in plasma could be a potential marker of oral cancer. Oral Dis..

[25] Liu C.J., Lin S.C., Yang C.C., Cheng H.W., Chang K.W. (2012). Exploiting salivary miR-31 as a clinical biomarker of oral squamous cell carcinoma. Head Neck.

[26] Tiberio P., Callari M., Angeloni V., Daidone M.G., Appierto V. (2015). Challenges in using circulating miRNAs as cancer biomarkers. Biomed Res. Int..

[27] He Y., Lin J., Kong D., Huang M., Xu C., Kim T.K., Etheridge A., Luo Y., Ding Y., Wang K. (2015). Current State of Circulating MicroRNAs as Cancer Biomarkers. Clin. Chem..

[28] Kirschner M.B., Kao S.C., Edelman J.J., Armstrong N.J., Vallely M.P., van Zandwijk N., Reid G. (2011). Haemolysis during sample preparation alters microRNA content of plasma. PLoS ONE.

[29] Moldovan L., Batte K.E., Trgovcich J., Wisler J., Marsh C.B., Piper M. (2014). Methodological challenges in utilizing miRNAs as circulating biomarkers. J. Cell Mol. Med..

[30] Yan Y., Wang X., Veno M.T., Bakholdt V., Sorensen J.A., Krogdahl A., Sun Z., Gao S., Kjems J. (2017). Circulating miRNAs as biomarkers for oral squamous cell carcinoma recurrence in operated patients. Oncotarget.

[31] Yang C.J., Shen W.G., Liu C.J., Chen Y.W., Lu H.H., Tsai M.M., Lin S.C. (2011). miR-221 and miR-222 expression increased the growth and tumorigenesis of oral carcinoma cells. J. Oral Pathol. Med..

[32] Zhou L., Jiang F., Chen X., Liu Z., Ouyang Y., Zhao W., Yu D. (2016). Downregulation of miR-221/222 by a microRNA sponge promotes apoptosis in oral squamous cell carcinoma cells through upregulation of PTEN. Oncol. Lett..

[33] Liu X., Yu J., Jiang L., Wang A., Shi F., Ye H., Zhou X. (2009). MicroRNA-222 regulates cell invasion by targeting matrix metalloproteinase 1 (MMP1) and manganese superoxide dismutase 2 (SOD2) in tongue squamous cell carcinoma cell lines. Cancer Genom. Proteom..

[34] Jiang F., Zhao W., Zhou L., Liu Z., Li W., Yu D. (2014). MiR-222 targeted PUMA to improve sensitization of UM1 cells to cisplatin. Int. J. Mol. Sci..

[35] Jiang F., Zhao W., Zhou L., Zhang L., Liu Z., Yu D. (2014). miR-222 regulates the cell biological behavior of oral squamous cell carcinoma by targeting PUMA. Oncol. Rep..

[36] Yu Z.Y., Bai Y.N., Luo L.X., Wu H., Zeng Y. (2013). Expression of microRNA-150 targeting vascular endothelial growth factor-A is downregulated under hypoxia during liver regeneration. Mol. Med. Rep..

[37] Naruse T., Kawasaki G., Yanamoto S., Mizuno A., Umeda M. (2011). Immunohistochemical study of VEGF expression in oral squamous cell carcinomas: Correlation with the mTOR-HIF-1alpha pathway. Anticancer Res.

[38] Roy R., Singh R., Chattopadhyay E., Ray A., Sarkar N.D., Aich R., Paul R.R., Pal M., Roy B. (2016). MicroRNA and target gene expression based clustering of oral cancer, precancer and normal tissues. Gene.

[39] Brase J.C., Johannes M., Schlomm T., Falth M., Haese A., Steuber T., Beissbarth T., Kuner R., Sultmann H. (2011). Circulating miRNAs are correlated with tumor progression in prostate cancer. Int. J. Cancer.

[40] Zhu C., Ren C., Han J., Ding Y., Du J., Dai N., Dai J., Ma H., Hu Z., Shen H. (2014). A five-microRNA panel in plasma was identified as potential biomarker for early detection of gastric cancer. Br. J. Cancer.

[41] Cabibi D., Caruso S., Bazan V., Castiglia M., Bronte G., Ingrao S., Fanale D., Cangemi A., Calo V., Listi A. (2016). Analysis of tissue and circulating microRNA expression during metaplastic transformation of the esophagus. Oncotarget.

[42] Jo P., Azizian A., Salendo J., Kramer F., Bernhardt M., Wolff H.A., Gruber J., Grade M., Beissbarth T., Ghadimi B.M. (2017). Changes of Microrna Levels in Plasma of Patients with Rectal Cancer during Chemoradiotherapy. Int. J. Mol. Sci..

[43] Molina-Pinelo S., Suarez R., Pastor M.D., Nogal A., Marquez-Martin E., Martin-Juan J., Carnero A., Paz-Ares L. (2012). Association between the miRNA signatures in plasma and bronchoalveolar fluid in respiratory pathologies. Dis. Markers.

[44] Brunet Vega A., Pericay C., Moya I., Ferrer A., Dotor E., Pisa A., Casalots A., Serra-Aracil X., Oliva J.C., Ruiz A. (2013). microRNA expression profile in stage III colorectal cancer: Circulating miR-18a and miR-29a as promising biomarkers. Oncol. Rep..

[45] Pigati L., Yaddanapudi S.C., Iyengar R., Kim D.J., Hearn S.A., Danforth D., Hastings M.L., Duelli D.M. (2010). Selective release of microRNA species from normal and malignant mammary epithelial cells. PLoS ONE.

[46] Shiah S.G., Shieh Y.S., Chang J.Y. (2016). The Role of Wnt Signaling in Squamous Cell Carcinoma. J. Dent. Res..

[47] Makarev E., Schubert A.D., Kanherkar R.R., London N., Teka M., Ozerov I., Lezhnina K., Bedi A., Ravi R., Mehra R. (2017). In silico analysis of pathways activation landscape in oral squamous cell carcinoma and oral leukoplakia. Cell Death Discov..

[48] Kroh E.M., Parkin R.K., Mitchell P.S., Tewari M. (2010). Analysis of circulating microRNA biomarkers in plasma and serum using quantitative reverse transcription-PCR (qRT-PCR). Methods.

