# Supplementary material for: A Three–MicroRNA Signature as a Potential Biomarker for the Early Detection of Oral Cancer
Source: Int J Mol Sci. 2018 Mar 5;19(3):758. doi: 10.3390/ijms19030758 (PMC5877619; doi:10.3390/ijms19030758)
Supplement: Supplementary file 1 [file ijms-19-00758-s001.pdf]

Supplementary information

1. Materials and Methods

1.1 Extraction of RNA from plasma

Blood was centrifuged at 3000 g for 10 min to separate plasma and stored at −80°C. Hemolysis was monitored based on the optical density at 414 nm [1]. For small RNA sequencing, equal volumes of each individual sample were mixed to obtain a pooled sample. Total RNA was extracted from 3 ml of pooled plasma using a combined version of phenol–chloroform extraction followed by column purification. RNA concentration was assessed using the Qubit RNA Assay Kit (Life Technologies). For individual qRT-PCR assays, 200 μl of plasma was extracted with miRNeasy Serum/Plasma Kit (Qiagen) and *Caenorhabditis elegans* synthetic miR-39 was added to serve as spike-in control and to evaluate the predence of according to the manufacturer's instruction.

1.2 Data analysis for small RNA sequencing

Reads of small RNA-seq were trimmed 3'adapter and quality pre-processed using cutadapt v1.9.1 [2] and FASTX-Toolkit v0.0.13.2 (FASTQ/A short-reads pre-processing tools, http://hannonlab.cshl.edu/fastx_toolkit/), respectively. The reads with Phred quality score ≥ 20 and longer than 18 nucleotides were retained. ncPRO-seq [3] package (version 1.6.1) were used to qualitative small RNA-seq, include reads length distribution, percentage of reads alignment in the reference genome, and proportion of small RNA via bowtie [4] v1.1.2 (parameter: -v1 -a -m20 --best --strata --nomaqround -f -y). The UCSC reference genome (hg38), miRBase v21, UCSC refGene, RFam v11.0, and UCSC repeatMasks (hg19) were employed for annotation of reads that could be mapped to genome. miRDeep2 [5] package (version 2.0.0.5) were used to quantify the miRNA profiles that employed the same parameter of bowtie as ncPRO-seq. The reads per million (RPM) were employed to normalize reads count in each sample.

1.3 Risk score analysis

For the correlation of combined miRNA with OL or OSCC risk, each patient was assigned a risk score function (RSF). The risk function (RSF) for patient i was calculated using the following formula:

$$RSF_i = \sum_{j=1}^{k}(W_j \times S_{ij})$$

Here, the score (S*ij*) of miRNA *j* on patient i was weighted by W*j*, the regression coefficient estimated by univariate logistic regression models for each miRNA [6, 7]. Based on the risk scores, ROCs of combined miRNA panel were also generated. ROC curves of miRNA panel were generated based on the predicted probability (P) for each patient. P = Exp (combined miRNA panel)/ [1+Exp (combined miRA level)].

1.4 The Cancer Genome Atlas (TCGA) miRNA sequencing data analysis

To verify if the expression patterns of the identified miRNAs were consistent between plasma and solid tissue, we collected the miRNA expression profiles of solid tissue of head and neck cancer from The Cancer Genome Atlas (TCGA, http://cancergenome.nih.gov/) and compared them with those of plasma in our study. The miRNA sequencing data and patients' clinical information of TCGA Head-Neck Squamous Cell Carcinoma (HNSC) dataset (data version: 2016_01_28) were retrieved from the FireBrowse database (http://firebrowse.org/). The Level 3 miRNA sequencing data with normalized miRNA expressions were analyzed for further elucidating the relationship between miRNA expressions and clinical stages. Samples extracted from oral cavity, oral tongue, floor of

mouth, buccal mucosa, base of tongue or hard plate were selected according to the descriptions of "anatomic neoplasm subdivision" in clinical information. A total of 295 tumor samples and 32 adjacent normal samples were selected for further analysis.

1.5 Identification of miRNA-targets and functional annotation of target genes

To identify high confidence miRNA-Target Interaction (MTIs), the experimentally verified MTIs with strong evidence were collected using miRTarBase 6.0 [8]. We also predicted miRNA-targets using TargetScanHuman 7.0 and miRDB. The previous step of experimental collection and predicted approach collection generated a list of genes. Functional annotation tools DAVID [9] was employed to illustrate the biological regulation role from Gene Ontology [10] or KEGG pathway database [11]. In addition, ingenuity pathway analysis (IPA) software (Ingenuity Systems, Inc.) was applied to analyze the canonical pathways networks, and biological functions of identified miRNAs.

References:

1. Kirschner MB, Edelman JJ, Kao SC, Vallely MP, van Zandwijk N, Reid G. The Impact of Hemolysis on Cell-Free microRNA Biomarkers. Front Genet. 2013; 4: 94. doi: 10.3389/fgene.2013.00094.
2. Martin M. Cutadapt removes adapter sequences from high-throughput sequencing reads. EMBnetjournal. 2011; 17: 10. doi: 10.14806/ej.17.1.200.
3. Chen CJ, Servant N, Toedling J, Sarazin A, Marchais A, Duvernois-Berthet E, Cognat V, Colot V, Voinnet O, Heard E, Ciaudo C, Barillot E. NcPRO-seq: A tool for annotation and profiling of ncRNAs in sRNA-seq data. Bioinformatics. 2012; 28: 3147-9. doi: 10.1093/bioinformatics/bts587.
4. Langmead B, Trapnell C, Pop M, Salzberg SL. Ultrafast and memory-efficient alignment of short DNA sequences to the human genome. Genome Biology. 2009; 10: R25. doi: 10.1186/gb-2009-10-3-r25.
5. Friedländer MR, MacKowiak SD, Li N, Chen W, Rajewsky N. MiRDeep2 accurately identifies known and hundreds of novel microRNA genes in seven animal clades. Nucleic Acids Research. 2012; 40: 37-52. doi: 10.1093/nar/gkr688.
6. Kroh EM, Parkin RK, Mitchell PS, Tewari M. Analysis of circulating microRNA biomarkers in plasma and serum using quantitative reverse transcription-PCR (qRT-PCR). Methods. 2010; 50: 298-301. doi: 10.1016/j.ymeth.2010.01.032.
7. Zhu C, Ren C, Han J, Ding Y, Du J, Dai N, Dai J, Ma H, Hu Z, Shen H, Xu Y, Jin G. A five-microRNA panel in plasma was identified as potential biomarker for early detection of gastric cancer. British Journal of Cancer. 2014; 110: 2291-9. doi: 10.1038/bjc.2014.119.
8. Chou CH, Chang NW, Shrestha S, Hsu SD, Lin YL, Lee WH, Yang CD, Hong HC, Wei TY, Tu SJ, Tsai TR, Ho SY, Jian TY, et al. miRTarBase 2016: Updates to the experimentally validated miRNA-target interactions database. Nucleic Acids Research. 2016; 44: D239-D47. doi: 10.1093/nar/gkv1258.
9. Huang DW, Sherman BT, Lempicki RA. Systematic and integrative analysis of large gene lists using DAVID bioinformatics resources. Nature Protocols. 2008; 4: 44-57. doi: 10.1038/nprot.2008.211.
10. Consortium TGO. Gene ontologie: Tool for the unification of biology. Nature Genetics. 2000; 25: 25-9. doi: 10.1038/75556.Gene.
11. Ogata H, Goto S, Sato K, Fujibuchi W, Bono H, Kanehisa M. KEGG: Kyoto encyclopedia of genes and genomes. Nucleic Acids Research. 1999; 27: 29-34. doi: 10.1093/nar/27.1.29.

2. Figures and Tables

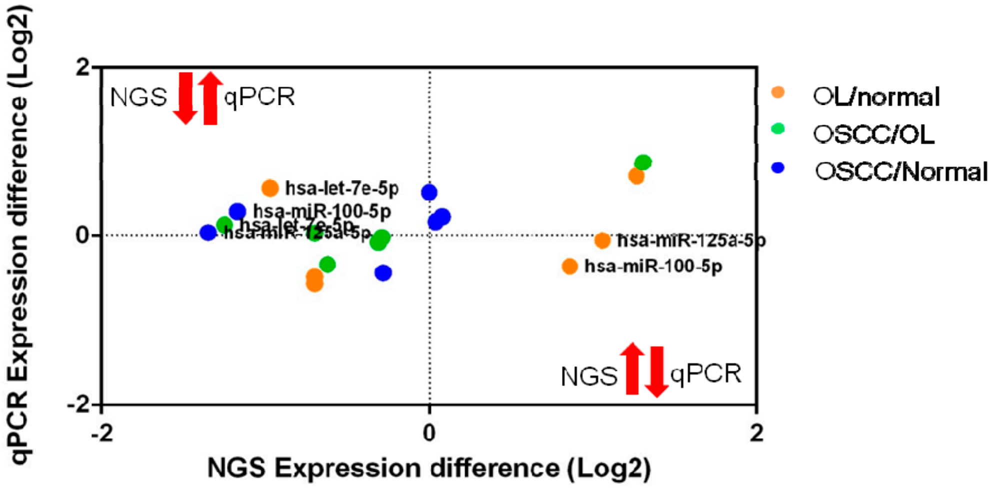

Supplementary Figure S1. qPCR confirmation of differentially expressed miRNAs identified by NGS**.** Each dot represents the differentially expressed miRNAs between specified groups. Inconsistent results between NGS and qPCR are shown.

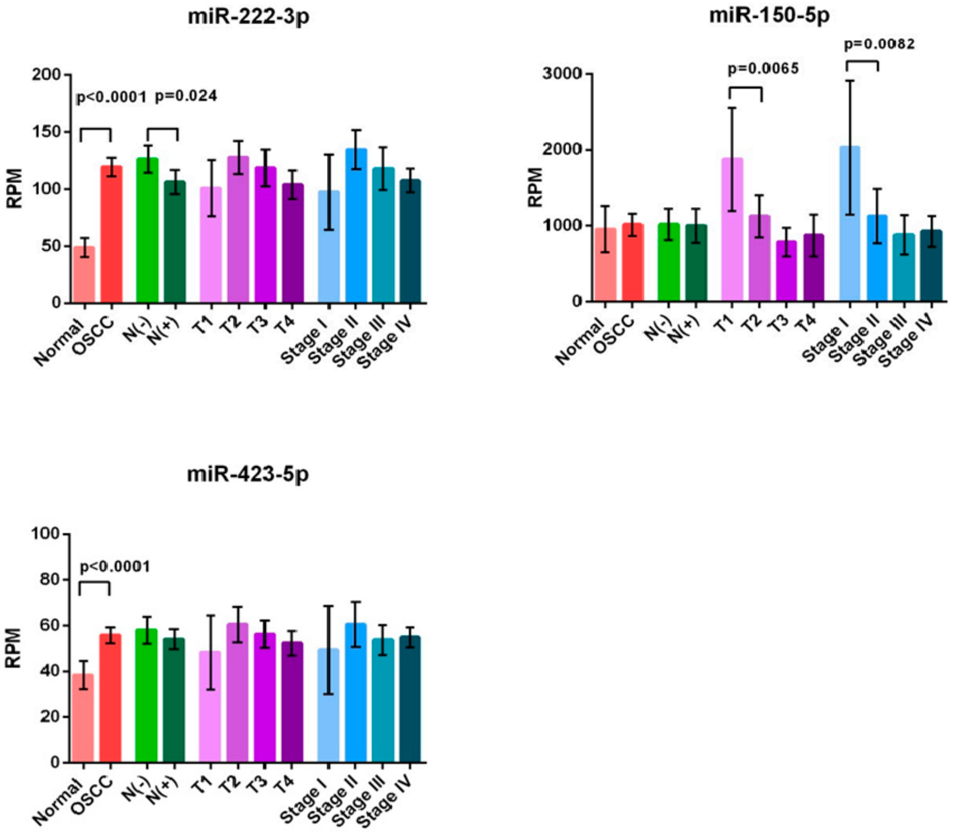

Supplementary Figure S2. TCGA analysis of identified miRNAs. TCGA data set from a total of 295 tumor samples and 32 adjacent normal samples were analyzed to compare the miRNA abundance among groups.

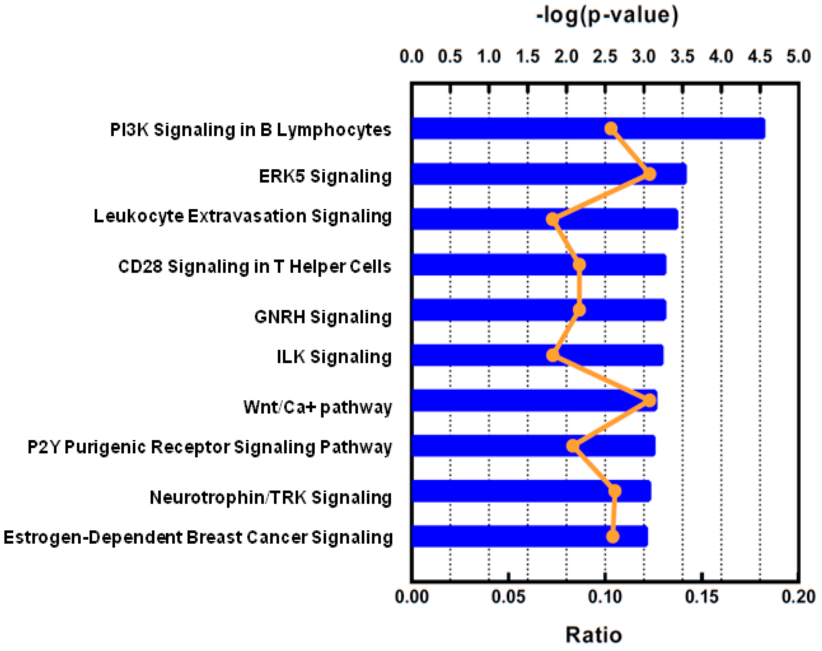

Supplementary Figure S3. The top 10 most enriched pathway by IPA analysis. Pathways with average z score > 2 or < -2 and –log (p-value) > 1.301 were included.

Supplementary Table S1. Ct values and gene expression stability of candidate reference genes evaluated by different methods

Variables	miR-130b-3p	miR221-3p	miR-101-3p	miR-16-5p
Ct values of all samples				
min	21.56	20.12	18.61	17.15
max	23.69	22.67	21.68	21.90
mean±SD	22.61±0.57	21.36±0.57	20.44±0.87	20.42±1.09
Ct values of different groups				
Controls	22.79±0.45	21.71±0.53	20.46±0.46	20.39±0.76
OLP	22.60±0.68	21.38±0.47	19.81±0.97	20.18±1.28
OSCC	22.47±0.51	21.07±0.56	21.13±0.40	20.71±1.11
p value[a]	0.492	0.133	0.001	0.469
Stability (rank)[b]				
DeltaCT	0.890 (1)	1.013 (3)	0.913 (2)	1.098 (4)
BestKeeper	0.445 (2)	0.426 (1)	0.668 (3)	0.824 (4)
NormFinder	0.491 (1)	0.792 (3)	0.519 (2)	0.924 (4)
Genorm	0.710 (1)[c]	0.710 (1)[c]	0.859 (3)	0.979 (4)
Recommended ranking	1.189 (1)	1.732 (2)	2.449 (3)	4.000 (4)

a: Kruskal–Wallis test
b: The smaller value of gene stability and rank indicates the more stable gene.
c: A combination of these two genes.

Supplementary Table S2. Top diseases and bio functions enriched with the targetome of three miRNAs

Name	No. of genes	P Value
**Diseases and disorders**		
Cancer	493	1.94E-03 - 2.45E-11
Organismal Injury and Abnormalities	500	1.94E-03 - 2.45E-11
Gastrointestinal Disease	443	1.94E-03 - 6.22E-11
Hepatic System Disease	244	1.36E-03 - 2.16E-09
Reproductive System Disease	320	1.40E-03 - 1.08E-08
**Molecular and Cellular Functions**		
Gene Expression	121	1.66E-03 - 2.81E-15
Cellular Growth and Proliferation	110	1.91E-03 - 1.44E-13
Cell Death and Survival	146	1.73E-03 - 1.44E-08
Cellular Development	113	1.62E-03 - 4.55E-08
Cell cycle	60	1.91E-03 - 8.97E-07
